# Innovations in Gastric Cancer Surgery During Early Minimally Invasive Era and Future Perspectives

**DOI:** 10.1002/ags3.70234

**Published:** 2026-05-10

**Authors:** Reut El‐On, Young‐Woo Kim

**Affiliations:** ^1^ Center for Gastric Cancer National Cancer Center Goyang Korea; ^2^ Division of Thoracic Surgery, Department of Surgery Brigham and Women's Hospital, Harvard Medical School Boston Massachusetts USA; ^3^ National Cancer Center Graduate School of Cancer Science and Policy Goyang Korea

**Keywords:** artificial intelligence, clinical trial, gastrectomy, gastric cancer, innovation, laparoscopy, lymphadenectomy, minimally invasive surgery, robotics, surgical quality

## Abstract

The landscape of gastric cancer surgery has undergone remarkable transformation, evolving from traditional open procedures to the modern era of live‐streamed minimally invasive operations. This evolution owes much to pioneering surgeons who have continued to expand the boundaries of innovation. Over recent decades, the field has progressed from the introduction of laparoscopic techniques to the refinement of lymphadenectomy, while surgeons have simultaneously integrated insights from tumor biology, advanced instrumentation, and data analytics to improve decision‐making and precision. Progress has been fueled by extensive clinical experience and collaborative multicenter trials that provide evidence‐based guidance for clinical practice. With continuing revelations in tumor biology and the emergence of artificial intelligence, new horizons for surgical innovation are opening. At the center of this transformative journey stands the innovative surgeon, driven by passion, guided by data, and steadfast in the commitment to patient safety and quality of life. The surgeon of the future will lead multicenter collaborations, integrating technology and biology to optimize outcomes and continually advance the standards of gastric cancer surgery.

## Graphical abstract

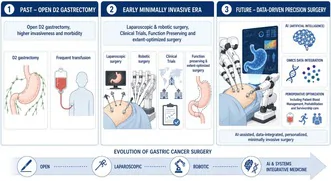

## 1 Introduction

Since the pioneering work of Theodor Billroth in the 19th century, gastric cancer surgery has witnessed tremendous progress. From the era of open procedures performed in traditional operating theaters to the current practice of minimally invasive surgeries that can be live‐streamed with real‐time commentary, the field has evolved continuously through the efforts of passionate surgeons and collaborative research. Advances in epidemiology and clinical science have enhanced understanding of risk factors, improved screening and early diagnosis, and increased the likelihood of cure for many patients.

A major milestone in the evolution of gastric cancer surgery was the validation of D2 lymphadenectomy with gastrectomy by renowned surgeons such as Kajitani, Okajima, and Maruyama, which established the oncologic safety and therapeutic purpose of extended lymph node dissection. Sasako's landmark randomized trial further shifted the focus toward patient survival and quality of life by demonstrating that prophylactic dissection did not improve outcomes and that more extensive surgery is not universally beneficial [1], thereby laying the foundation for the modern, evidence‐based approach to determining the extent of gastric cancer surgery.

Japanese surgeons have played pivotal roles in the subsequent development of minimally invasive gastric cancer surgery. In 1994, Kitano reported the first laparoscopic gastrectomy for gastric cancer, inspiring further investigation and broader adoption of laparoscopic techniques [2]. Not long thereafter, Hashizume performed the first robotic gastrectomy in 2002, initiating a new phase of technological integration in gastric surgery [3]. The period from the mid‐1990s to the mid‐2010s represents the early minimally invasive era in gastric cancer surgery—approximately two decades during which laparoscopic and early robotic techniques were systematically evaluated, refined, and gradually integrated into clinical practice, primarily in East Asian centers with high case volumes. Although many surgeons were initially hesitant to support these minimally invasive procedures because of concerns about safety, oncologic adequacy, and long‐term outcomes, accumulating evidence has now firmly established the efficacy and safety of minimally invasive approaches for appropriately selected patients.

### 1.1 International Collaboration and Knowledge Sharing

Meaningful innovation in gastric cancer surgery depends on active collaboration and knowledge‐sharing across institutions and countries. Japan, Korea, and China have long exemplified this spirit through joint research, consensus‐building, and live demonstration surgeries. When Uyama shared detailed operative videos of laparoscopic D2 dissections, the clarity and sophistication of these procedures astonished audiences and significantly advanced understanding of how minimally invasive surgery could replicate, and in some respects improve upon, traditional open techniques [4].

International teleconferences and live surgical transmissions further accelerate progress. The experience of performing minimally invasive gastric cancer surgery in real‐time dialogue with experts such as Shimizu illustrates the educational power of technology‐enabled collaboration. Such events provide substantial benefit even for highly experienced surgeons, creating a platform for shared learning, technical refinement, and harmonization of standards across centers with differing case volumes and resources [5].

### 1.2 Integration of Clinical Research and Surgical Innovation

The symbiotic relationship between clinical practice and research is central to sustainable surgical progress. Surgeons must remain open‐minded and curious, constantly questioning current practice, anticipating challenges, and proactively seeking solutions through meticulous study. The introduction of new surgical techniques, especially minimally invasive approaches, requires careful evaluation to address concerns about safety, feasibility, and oncologic outcomes.

When laparoscopic gastrectomy was introduced for early gastric cancer, a single‐center, single‐surgeon pilot study involving 164 patients was conducted [6]. This study demonstrated the safety of laparoscopic gastrectomy and highlighted postoperative improvements in patient quality of life. Building on these results, a larger multicenter Phase 2 trial was conducted in collaboration with eight centers to evaluate the feasibility of laparoscopic surgery for more advanced gastric cancer. This trial confirmed that laparoscopic approaches could be safely extended to a broader spectrum of disease when performed in experienced centers [7]. These initial studies provided the foundation for subsequent large multicenter randomized trials.

Evidence Based Evolution Through Multicenter Randomized Trial.

The establishment of minimally invasive approaches as standard treatment options has been driven by rigorous multicenter randomized controlled trials conducted primarily in East Asia. Table 1 summarizes landmark clinical trials that have shaped the evidence base for laparoscopic gastrectomy in Korea, Japan, and China.

**TABLE 1:** Landmark randomized controlled trials for laparoscopic gastrectomy in gastric cancer.

Trial	Country	Publication	Design	Primary endpoint	Key findings
KLASS‐01 [8]	Korea	2019	Phase 3 RCT	5 year DFS	LDG noninferior to ODG for EGC
KLASS‐02 [9]	Korea	2020	Phase 3 RCT	3 year RFS	LDG + D2 comparable to ODG for AGC
JCOG0912 [10]	Japan	2020	Phase 3 RCT	5 year RFS	LADG noninferior to ODG for stage I
JLSSG0901 [11]	Japan	2023	Phase 3 RCT	5 year RFS	LADG noninferior to ODG for AGC
CLASS‐01 [12]	China	2019	Phase 3 RCT	3 year DFS	LDG + D2 noninferior to ODG for AGC
CLASS‐02 [13]	China	2020	Phase 2 RCT	Morbidity and mortality	LTG comparable to OTG for stage I GC

Abbreviations: AGC, advanced gastric cancer; D2, D2 lymphadenectomy; DFS, disease‐free survival; EGC, early gastric cancer; GC, gastric cancer; LADG, laparoscopy‐assisted distal gastrectomy; LDG, laparoscopic distal gastrectomy; LTG, laparoscopic total gastrectomy; ODG, open distal gastrectomy; OTG, open total gastrectomy; RCT, randomized controlled trial; RFS, relapse‐free survival.

The KLASS (Korean Laparoendoscopic Gastrointestinal Surgery Study) group has systematically evaluated laparoscopic approaches over two decades, beginning with KLASS‐01, which demonstrated the noninferiority of laparoscopic distal gastrectomy to open surgery for early gastric cancer. KLASS‐02 extended these findings to locally advanced gastric cancer, confirming comparable relapse‐free survival and reduced complications with laparoscopic D2 lymphadenectomy. The JCOG (Japan Clinical Oncology Group) trials have paralleled these efforts, with JCOG0912 establishing the safety of laparoscopy‐assisted distal gastrectomy for stage I disease. China's CLASS (Chinese Laparoscopic Gastrointestinal Surgery Study) trials have contributed complementary evidence for both distal and total gastrectomy approaches. Together, these multicenter investigations transformed laparoscopic gastrectomy from an experimental technique to an evidence‐based standard of care.

For robotic gastrectomy, evidence is emerging but remains less mature. While comparative studies have shown that robotic gastrectomy is feasible and yields outcomes broadly comparable to laparoscopic approaches, including reduced blood loss and similar lymph node harvest, large‐scale randomized trials remain limited [14]. Ongoing multicenter trials in East Asia are expected to further clarify the role of robotic surgery in treatment guidelines.

### 1.3 Surgeon‐Driven Technological Development

In addition to evaluating new surgical approaches, surgeons play a critical role in driving the development of surgical technologies themselves. Beyond patient‐related factors, a holistic view of the surgical environment reveals many opportunities for improvement in visualization, ergonomics, and instrumentation. During the practice of laparoscopic surgery, the author identified significant limitations in conventional two‐dimensional endoscopic visualization, particularly in‐depth perception and structural recognition.

Motivated by these observations, collaboration with engineering development teams led to the creation of three‐dimensional laparoscopic endoscopes. By enhancing depth perception and emphasizing structural detail, this device improved the surgeon's ability to perform precise dissections and complex reconstructions. This example illustrates how surgeons can and should actively participate in technological innovation, using real‐world operative experience to guide the design of tools that directly impact safety, efficiency, and patient outcomes.

Cultivating this skill set should begin early in surgical education and be nurtured throughout a surgeon's career. Surgeons who engage in technology development and validation are well positioned to shape the future of practice and ensure that new devices and systems address clinically meaningful needs.

### 1.4 Global Skill Development and Standardization

The contributions of surgeons from Japan, China, and Korea have led to a paradigm shift in gastric cancer surgery, particularly in the adoption of minimally invasive techniques and function‐preserving procedures. However, the translation of these innovations to other regions can be challenging. Differences in case volume, resources, training infrastructure, and disease epidemiology may impede widespread implementation of complex procedures developed in high‐volume expert centers.

To bridge this gap, targeted initiatives are needed to enhance the skills and knowledge of surgeons worldwide. Structured mentoring and monitoring programs can provide longitudinal support, while visiting fellowships and hands‐on workshops facilitate the transfer of technical skills. Virtual training platforms, high‐fidelity simulation, and tele‐mentoring can further democratize access to education by allowing surgeons in low‐ and middle‐income settings to observe expert operations, receive feedback, and progressively adopt advanced techniques.

Leveraging these technologies to build global networks of expertise will be essential for broadening the impact of innovations developed in East Asia and ensuring more uniform standards of gastric cancer care worldwide.

The introduction of technically demanding innovations to other institutions or regions may also encounter additional barriers, including variability in surgeon experience, heterogeneity in perioperative support systems, differences in available instrumentation, and challenges in reproducing subtle technical details outside high‐volume centers. The safest dissemination strategy is therefore not simple publication alone, but structured implementation through mentoring, objective assessment of surgical proficiency, operative manuals, educational videos, monitoring, and ideally prospective clinical evaluation.

The most effective approach is to embed the introduction of innovative procedures within a clinical trial that incorporates a structured surgical quality assurance program. By combining objective assessment of surgical proficiency with standardized operative guidelines, procedure manuals, demonstration materials such as videos and step‐by‐step images, and validated evaluation forms, surgical technique can be standardized and disseminated more safely across centers [15].

### 1.5 Optimization and Personalization of Surgical Techniques

As certain procedures become standard practice, a new challenge emerges: optimization. One key area is reconstruction after proximal gastrectomy, where various anastomotic methods have been developed to improve function and reduce complications. Numerous innovative reconstructive techniques have been proposed in Japan and Korea, encouraging other surgeons to learn, adapt, and refine their practice.

The SPADE technique, for example, employs double‐suture anchoring between the posterior wall of the esophagus and the anterior wall of the stomach while creating a flap and pseudo‐fornix, thereby improving anti‐reflux function and reducing postoperative complications [16]. This technique exemplifies ongoing refinement in reconstructive surgery after proximal gastrectomy. *Similarly*, the OFFROAD (Omental Free Flap Reinforcement on Anastomosis and Dissected area) technique utilizes remaining omental tissue to reinforce anastomoses and dissection sites, with the goals of reducing postoperative morbidity and minimizing adhesions. Preliminary studies have demonstrated that this approach does not significantly increase operative time or complication rates and may mitigate the severity of leaks when they occur [17].

These institutional innovations are promising but require broader validation before widespread adoption and underscore the iterative nature of surgical progress. The ongoing ADDICT trial further exemplifies the pursuit of surgical optimization by evaluating the appropriate extent of lymph node dissection, such as D1+ versus D2, for advanced gastric cancer [18], with the aim of improving both surgical outcomes and postoperative quality of life.

### 1.6 Function‐Preserving Surgery and Tumor Biology

Another essential question is which patient should receive which specific type of surgery. For example, it remains an area of active investigation whether subtotal gastrectomy is always necessary early gastric cancer in the middle third of the stomach or whether early gastric cancer requires the same surgical extent as advanced disease. Integrating detailed knowledge of tumor behavior with tailored surgical techniques has led to more individualized function‐preserving approaches. One such approach is middle segmental gastrectomy, an evolution of pylorus‐preserving gastrectomy, designed to maintain gastric function, reduce anastomotic tension, and remain oncologically sound for tumors located in the middle third of the stomach [19].

Inspired by data from melanoma trials, the concept of sentinel node navigation surgery is also being explored in gastric cancer. Kitagawa and others have reported feasibility studies showing that sentinel node mapping can identify relevant lymphatic basins in early gastric cancer [20]. The SENORITA group, led by Ryu and involving multiple centers, has conducted trials to refine indications and techniques for sentinel node navigation surgery in this context [21]. These studies collectively suggest that incorporating biologic information from sentinel nodes can enable more tailored, less extensive resections in carefully selected patients.

Further understanding of tumor biology, molecular characteristics, and metastatic patterns will facilitate more precise patient selection and surgical planning. Such insights may ultimately support patient‐specific screening protocols and genuinely individualized surgical strategies that improve oncologic control while preserving function and health‐related quality of life.

### 1.7 Perioperative Optimization and Patient Blood Management

The surgeon's responsibility for optimizing patient outcomes begins well before entering the operating room. Careful management of comorbidities and attention to seemingly minor abnormalities can substantially influence prognosis. Anemia is one of the most important factors to address preoperatively, as suboptimal hemoglobin levels can worsen postoperative recovery, increase complication rates, and negatively affect long‐term outcomes.

Surgeons should incorporate evidence‐based strategies to correct anemia and minimize perioperative blood loss. A multicenter, 7 year clinical trial has demonstrated that intravenous ferric carboxymaltose can significantly improve hemoglobin levels and reduce the need for transfusion in surgical patients [22]. In parallel, the concept of patient blood management (PBM), supported by the World Health Organization, emphasizes evidence‐based transfusion thresholds, meticulous intraoperative hemostasis, and preservation of the patient's own blood. Implementing PBM protocols can improve recovery, shorten hospital stays, and reduce transfusion‐related risks [23].

Integrating these perioperative strategies into routine practice reflects the broader movement toward holistic, patient‐centered care, in which surgical technique, perioperative medicine, and long‐term survivorship are considered together.

### 1.8 Technology, Data, and the Emergence of Artificial Intelligence

The future of gastric cancer surgery lies at the intersection of technology, biology, and precision surgery. Endoscopic resection has expanded rapidly in East Asia and now manages a substantial proportion of early gastric cancers. This evolution underscores the need for advanced, surgeon‐oriented instruments that support both endoscopic and laparoscopic techniques, ensuring that the operating surgeon retains full control over resection strategy while benefiting from enhanced visualization and precision.

Over the next decade, we anticipate the generation of vast amounts of proteomic, genomic, and radiomic data, which will need to be translated into clinically actionable information. Such data have the potential to guide individualized treatment plans, including the choice and extent of surgery, the use of neoadjuvant or adjuvant therapies, and postoperative surveillance strategies. Technological innovation will support these advances through tools such as flexible robotic platforms (e.g., K‐Flex) [24], improved imaging modalities, and preoperative modeling using holograms and three‐dimensional printing to plan complex resections.

Over the next decade, vast amounts of genomic, proteomic, and radiomic data are expected to become increasingly available and will need to be translated into clinically actionable information. Integration of such data may support individualized treatment plans, including preventive strategies, the choice and extent of surgery, perioperative therapies, and postoperative surveillance [25].

Artificial intelligence is progressively transitioning from conceptual promise to practical clinical application in gastric cancer surgery. Several domains have demonstrated concrete evidence of utility.

Diagnostic and detection applications: AI‐assisted endoscopy systems using deep learning algorithms have demonstrated improved detection rates of early gastric cancer lesions, reduced operator‐dependent variability and potentially enabling earlier diagnosis [26]. Computer‐aided detection systems can analyze endoscopic images in real time, highlight suspicious areas, and support less experienced endoscopists.

Intraoperative guidance and surgical phase recognition: AI‐based computer vision systems are being developed to provide real‐time anatomical recognition during laparoscopic and robotic gastrectomy. Recent studies have demonstrated models capable of recognizing surgical phases, identifying critical anatomical structures such as lymph node stations, major vessels, and dissection planes, and providing intraoperative feedback to enhance safety and accuracy [27]. Such systems may support surgical education, enable real‐time decision support, and facilitate automated operative documentation.

Perioperative risk prediction and outcome modeling: Machine learning algorithms integrating preoperative clinical, laboratory, and imaging data have shown promise for predicting survival and postoperative complications in gastric cancer patients [28]. AI‐driven frailty assessment and risk stratification tools may support individualized prehabilitation planning and perioperative optimization, thereby helping identify patients who may benefit from intensified preparation or alternative treatment strategies [29].

Current limitations and obstacles: Despite these advances, significant barriers remain before widespread clinical adoption is possible. Many AI applications in gastric cancer surgery are still based on relatively small, single‐center datasets with limited external validation. Important concerns include data privacy, algorithmic interpretability, medico‐legal responsibility, and integration into existing clinical workflows [30]. Although autonomous robotic systems have demonstrated highly precise performance in selected experimental tasks, they remain far from safe independent deployment in complex oncologic surgery, where adaptability, judgment, and ethical responsibility remain fundamentally human [31]. AI should therefore be developed as a tool to augment, rather than replace, surgeon expertise, and its implementation should be guided by robust ethical frameworks, transparent validation, and patient safety [32].

## 2 Conclusion: Future Perspectives and the Role of the Surgeon

As AI and other technologies continue to evolve, a collaborative model in which surgeons and intelligent systems work together is likely to emerge. In this model, AI systems may assist with case selection, operative planning, intraoperative guidance, and postoperative monitoring, while surgeons provide clinical context, interpret unexpected findings, and make nuanced decisions that incorporate patient preferences and values.

Innovation in gastric cancer surgery will continue to depend on surgeons who are curious, data‐driven, and committed to patient safety and quality of life. These surgeons will lead multicenter collaborations, design and interpret clinical trials, and partner with engineers and basic scientists to create the next generation of surgical tools. They will also play crucial roles in education, mentoring, and global capacity‐building to ensure that advances benefit patients worldwide, not only those treated in high‐volume centers.

Ultimately, it is through surgical innovation grounded in clinical experience, collaborative research, and surgeon‐directed technological development that the field of gastric cancer surgery will reach new heights. By embracing this integrated approach, surgeons can transform care for future generations of patients.

## Author Contributions

**Young‐Woo Kim:** conceptualization, investigation, supervision, writing – original draft, writing – review and editing. **Reut El‐On:** investigation, writing – original draft, writing – review and editing.

## Funding

The authors have nothing to report.

## Conflicts of Interest

The authors declare no conflicts of interest.

## Data Availability

Data sharing not applicable to this article as no datasets were generated or analysed during the current study.
